# Matched cohort study of germline *BRCA* mutation carriers with triple negative breast cancer in brightness

**DOI:** 10.1038/s41523-021-00349-y

**Published:** 2021-11-11

**Authors:** Otto Metzger-Filho, Katharine Collier, Sarah Asad, Peter J. Ansell, Mark Watson, Junu Bae, Mathew Cherian, Joyce O’Shaughnessy, Michael Untch, Hope S. Rugo, Jens B. Huober, Mehra Golshan, William M. Sikov, Gunter von Minckwitz, Priya Rastogi, Lang Li, Lijun Cheng, David Maag, Norman Wolmark, Carsten Denkert, W. Fraser Symmans, Charles E. Geyer, Sibylle Loibl, Daniel G. Stover

**Affiliations:** 1 Medical Oncology, Dana-Farber/Partners CancerCare, Boston, MA USA; 2 Department of Medicine, The Ohio State University College of Medicine, Columbus, OH USA; 3 Division of Medical Oncology, The Ohio State University Comprehensive Cancer Center, Columbus, OH USA; 4 AbbVie, North Chicago, IL USA; 5 Washington University School of Medicine, St. Louis, MO USA; 6 Baylor University Medical Center, Texas Oncology, U.S. Oncology, Dallas, TX USA; 7 Helios Klinikum Berlin-Buch, Berlin, Germany; 8 University of California, San Francisco, San Francisco, CA USA; 9 University Medical Center Ulm, Ulm, Germany; 10 Department of Surgery, Yale Cancer Center, New Haven, CT USA; 11 Women and Infants Hospital of Rhode Island, Providence, RI USA; 12 German Breast Group, Neu-Isenburg, Germany; 13 University of Pittsburgh Medical Center Hillman Cancer Center, Pittsburgh, PA USA; 14 Department of Biomedical Informatics, The Ohio State University, Columbus, OH USA; 15 Allegheny General Hospital, Pittsburgh, PA USA; 16 Institute of Pathology, Philipps-University Marburg and University Hospital Marburg (UKGM), Marburg, Germany; 17 University of Texas, MD Anderson Cancer Center, Houston, TX USA; 18 Virginia Commonwealth University Massey Cancer Center, Richmond, VA USA; 19 Present Address: Houston Methodist, Houston, TX USA

**Keywords:** Breast cancer, Breast cancer

## Abstract

In the BrighTNess trial, carboplatin added to neoadjuvant chemotherapy (NAC) was associated with increased pathologic complete response (pCR) rates in patients with stage II/III triple-negative breast cancer (TNBC). In this matched cohort study, cases with a germline *BRCA1/2* mutation (gBRCA; *n* = 75) were matched 1:2 with non-gBRCA controls (*n* = 150) by treatment arm, lymph node status, and age to evaluate pCR rates and association of benefit from platinum/PARP inhibitors with validated RNA expression-based immune, proliferation, and genomic instability scores among gBRCA with the addition of carboplatin ± veliparib to NAC. Among the well-matched cohorts, odds of pCR were not higher in gBRCA cancers who received standard NAC with carboplatin (OR 0.24, 95% CI [0.04-1.24], p = 0.09) or with carboplatin/veliparib (OR 0.44, 95% CI [0.10-1.84], *p* = 0.26) compared to non-gBRCA cancers. Higher PAM50 proliferation, GeparSixto immune, and CIN70 genomic instability scores were each associated with higher pCR rate in the overall cohort, but not specifically in gBRCA cases. In this study, gBRCA carriers did not have higher odds of pCR than non-gBRCA controls when carboplatin ± veliparib was added to NAC, and showed no significant differences in molecular, immune, chromosomal instability, or proliferation gene expression metrics.

## Introduction

In localized triple-negative breast cancer (TNBC), neoadjuvant chemotherapy (NAC) allows for assessment of pathologic response to chemotherapy. Pathologic complete response (pCR) is associated with improved overall survival compared to residual disease^1–4^. To improve pCR rates, studies have attempted to add additional agents to the anthracycline plus taxane NAC backbone. Three studies have shown an improvement in pCR rate with the addition of carboplatin: GeparSixto^5^, CALGB 40603^6^, and I-SPY2^7^. Expanding on I-SPY2, BrighTNess was a phase III, multicenter, international, randomized, double-blind, placebo-controlled clinical trial, which enrolled 634 patients with stage II/III TNBC 2:1:1 to NAC with: (A) paclitaxel plus carboplatin plus veliparib followed by doxorubicin plus cyclophosphamide (TCV-AC), (B) paclitaxel plus carboplatin followed by doxorubicin plus cyclophosphamide (TC-AC), or (C) paclitaxel followed by doxorubicin plus cyclophosphamide (T-AC). In BrighTNess, the rate of pCR was higher in the TCV-AC (53%) and TC-AC (58%) arms, compared to T-AC (31%)^8^.

*BRCA* mutations result in impaired DNA damage repair by homologous recombination, which leads to sensitivity to PARP inhibitors and platinum chemotherapy in metastatic TNBC, as seen in the OlympiAD and TNT trials, respectively^9,10^. In the BrighTNess trial, 14–16% of patients in each arm had a confirmed deleterious germline *BRCA1/2* mutation^8^. Interestingly, in the subgroup analysis of patients with germline *BRCA* mutations there was no difference in pCR between *BRCA* mutated and wildtype patients overall: 51% of patients with a *BRCA* mutation (g*BRCA*), achieved a pCR, compared to 48% of patients that did not harbor a *BRCA* mutation (non-g*BRCA*)^8^. There was a trend among patients with germline *BRCA* mutations toward higher pCR rates compared to T-AC alone (41%) with the addition of carboplatin to paclitaxel (50%), and a further trend toward higher pCR as veliparib was added to carboplatin and paclitaxel (57%)^8^. In non-g*BRCA* patients, pCR rates were 29% for T-AC alone, 59% with the addition of carboplatin, and 53% with the addition of veliparib with carboplatin^8^.

Recently, we published correlative genomic analysis of gene expression for 482 of 634 patients enrolled in BrighTNess^11^. We found that in multivariable analysis, proliferation and immune signatures were independently associated with pCR but that carboplatin benefit was not significantly different in basal-like vs. non-basal subgroups. Exploratory gene expression immune analyses suggested that tumors with higher inferred CD8^+^ T-cell infiltration may receive greater benefit with the addition of carboplatin^11^.

Based on these data, we hypothesized that patients with *BRCA* mutated TNBC in the BrighTNess trial may benefit differentially from the addition of carboplatin or veliparib to NAC and may have distinct immune gene expression profiles. To evaluate this, we analyzed the association of pCR rate with treatment arm in a matched cohort study of patients with germline *BRCA1/2* (gBRCA) mutations compared to non-gBRCA controls. Using RNA sequencing of pre-treatment biopsies from the BrighTNess trial, we compared molecular subtype (PAM50 and TNBCtype), a measure of tumor proliferation (PAM50 proliferation score), a measure of chromosomal instability (CIN70), and measures of infiltrating immune cells (GeparSixto immune activation signature and relative immune cell abundance by TIMER) between gBRCA and non-gBRCA patients. Then, we determined if any of these measures were associated with pCR, which would indicate a subset of patients that would potentially benefit from the addition of carboplatin or veliparib.

## Results

### Matched cohort characteristics

There were 75 *BRCA1/2* mutated (gBRCA) cases and 150 non-gBRCA matched controls. *BRCA* cases and controls did not differ significantly by intentionally matched characteristics (planned treatment arm, lymph node status, age), nor by doxorubicin and cyclophosphamide (AC) administration schedule, Eastern Cooperative Oncology Group performance status, PAM50 subtype, TNBCtype, or rate of pCR (Table 1). Furthermore, *BRCA* cases did not differ from controls in terms of PAM50 proliferation score, GeparSixto immune activation signature score, CIN70 score, or TIMER-based relative abundance of tumor-infiltrating B cells, CD4 + T-cells, CD8 + T-cells, neutrophils, macrophages, or dendritic cells (Table 2).

**Table 1:** Cohort characteristics.

Characteristic	*BRCA* cases (*N* = 75)		Matched Controls (*N* = 150)		Chi-square
	No.	%	No.	%	*p*-value
Planned arm^1^					1.00
A	39	52.0	78	52.0	
B	18	24.0	36	24.0	
C	18	24.0	36	24.0	
Lymph node stage^1^					1.00
N0	41	54.7	82	54.7	
N1-2	34	45.3	68	45.3	
Age range^1^ (years)					1.00
Under 40	35	46.7	70	46.7	
41–50	21	28.0	42	28.0	
51–60	15	20.0	30	20.0	
61–70	4	5.3	8	5.3	
Planned administration schedule					0.57
Every 2 weeks	38	50.7	82	54.7	
Every 3 weeks	37	49.3	68	45.3	
ECOG status at baseline					0.63
0	69	92.0	135	90.0	
1	6	8.0	15	10.0	
PAM50 subtype					0.29
Basal	61	81.3	130	86.7	
Nonbasal	14	18.7	20	13.3	
TNBC subtype					0.90
BL1	18	24.0	29	19.3	
BL2	3	4.0	8	5.3	
IM	14	18.7	35	23.3	
LAR	2	2.7	8	5.3	
M	17	22.7	30	20.0	
MSL	8	10.7	16	10.7	
UNS	13	17.3	24	16.0	
Pathologic response					0.78
Residual disease	34	45.3	71	47.3	
Complete response (pCR)	41	54.7	79	52.7	

^1^Planned arm, lymph node stage, and age range were used to match controls to cases (2:1).

*ECOG* Eastern Cooperative Oncology Group, *No*. number, *pCR* pathologic complete response, *TNBC* triple-negative breast cancer, *BL1* basal-like 1, *BL2* basal-like 2, *IM* immunomodulatory, *LAR* luminal androgen receptor, *M* mesenchymal, *MSL* mesenchymal stem-like, *UNS* unselected.

**Table 2:** T-tests comparing proliferation, GeparSixto, and TIMER variables by *BRCA* status.

Variable	*BRCA* cases (*N* = 75)		Matched controls (*N* = 150)		T-test^1^
	Mean	Standard deviation	Mean	Standard deviation	*p*-value
Proliferation score	0.12	0.37	0.14	0.33	0.70
GeparSixto score	2.02	0.79	2.16	0.80	0.23
CIN70 score	3.84	0.62	3.80	0.51	0.63
B cells	9.61	0.78	9.77	0.93	0.21
CD4 T cells	12.18	1.00	12.35	1.14	0.28
CD8 T cells	20.55	1.27	20.60	1.22	0.77
Neutrophils	12.39	0.49	12.45	0.54	0.40
Macrophages	5.39	0.84	5.40	0.76	0.94
Dendritic cells	49.25	1.04	49.46	1.20	0.21

^1^Student’s t-test was used for variables that met the equal variance assumption (proliferation score, GeparSixto score, B cells, CD4 and CD8 T cells, neutrophils, macrophages, and dendritic cells). Welch’s *t*-test was used for CIN70.

### *BRCA* status and treatment response

We assessed interactions between treatment arm and *BRCA* status. Similar to the overall BrighTNess analysis the entire cohort of mutated and non-mutated patients had higher pCR rates with the addition of carboplatin with or without veliparib compared to T-AC alone (Table 3). Arm A, TCV-AC, (odds ratio [OR]: 4.55; 95% confidence interval [CI]: [1.89, 10.97]) and Arm B, TC-AC, (OR: 5.31; 95%CI: [1.92, 14.66]) had significantly higher odds of pCR compared to Arm C, T-AC; however, there were no significant interactions between treatment arm and *BRCA* status for the prediction of pCR (Table 3). The pCR rates by treatment arm and *BRCA* status are shown in Supplementary Table 1. When stratifying by *BRCA* status, we found that it was the non-gBRCA, not the gBRCA, patients who had higher odds of pCR with the addition of carboplatin with or without veliparib compared to T-AC alone (Supplementary Table 2).

**Table 3:** Logistic regression for pathologic complete response by *BRCA* status and treatment.

BRCA status and treatment	Odds Ratio	95% Confidence Interval	*p*-value
*BRCA* Case vs. Matched Control	2.40	(0.72,7.95)	0.15
Arm A vs. C	4.55	(1.89,10.97	0.0007
Arm B vs. C	5.31	(1.92,14.66)	0.001
*BRCA* Case vs. Matched Control *Arm A vs. C	0.44	(0.10,1.84)	0.26
*BRCA* Case vs. Matched Control *Arm B vs. C	0.24	(0.04,1.24)	0.09

Arm A: Paclitaxel, carboplatin, veliparib followed by doxorubicin and cyclophosphamide.

Arm B: Paclitaxel, carboplatin followed by doxorubicin and cyclophosphamide.

Arm B: Paclitaxel followed by doxorubicin and cyclophosphamide.

### Gene expression features of gBRCA and non-gBRCA TNBCs

PAM50 proliferation score, GeparSixto immune activation signature score, and CIN70 score independently predict pCR (*p* = 0.007, 0.007, and 0.003, respectively), but there was no significant interaction between the scores and *BRCA* status (Table 4). Specifically, higher PAM50 proliferation score (OR: 3.14; 95% CI: [1.36, 7.23]), higher GeparSixto immune activation signature (OR: 1.67; 95% CI: [1.15, 2.44]), and higher CIN70 score (OR: 2.15; 95% CI: [1.29, 3.58]) were associated with an increased odds of pCR (Table 4). PAM50 subtype was not significantly associated with pCR. The study was underpowered to assess for association of TNBCtype with pCR. We assessed the associations between the proliferation, GeparSixto, and CIN70 scores, and found that proliferation score and CIN70 score were significantly, positively correlated (Supplementary Fig. 1).

**Table 4:** Logistic regression for pathologic complete response by subtype and proliferation, GeparSixto, and CIN70 scores.

Subtype and scores	Odds Ratio	95% Confidence interval	*p*-value
PAM50 subtype			
Nonbasal vs. basal	0.77	(0.52,1.12)	0.17
*BRCA* Case vs. Matched Control	1.17	(0.80,1.71)	0.41
Nonbasal vs. basal*BRCA Case vs. Matched Control	1.16	(0.80,1.70)	0.44
Proliferation score			
Proliferation score	3.14	(1.36,7.23)	0.007
* BRCA* Case vs. Matched Control	1.13	(0.84,1.54)	0.41
Proliferation*BRCA Case vs. Matched Control	0.56	(0.24,1.30)	0.18
GeparSixto score			
GeparSixto score	1.67	(1.15,2.44)	0.007
* BRCA* Case vs. Matched Control	1.10	(0.48,2.49)	0.82
GeparSixto*BRCA Case vs. Matched Control	0.99	(0.68,1.45)	0.97
CIN70 score			
CIN70 score	2.15	(1.29,3.58)	0.003
* BRCA* Case vs. Matched Control	4.66	(0.65,33.55)	0.13
CIN70*BRCA Case vs. Matched Control	0.67	(0.40,1.12)	0.13

As an exploratory, unbiased approach to broadly interrogate diverse pathways involved in cancer and the immune microenvironment, we performed single sample gene set enrichment analysis (GSEA) using Hallmark and Immune Response In Silico (IRIS)^12^ gene sets. None of the gene sets demonstrated significant differences between *BRCA* cases and controls after multiple test correction and only seven gene sets demonstrated nominal (*p* < 0.10) association with *BRCA* cases: WNT/Beta-catenin signaling (Hallmark), two neutrophil gene sets (IRIS), and two memory B-cell gene sets (IRIS) (Supplementary Tables 3 and 4).

## Discussion

The role of carboplatin in NAC regimens for TNBC remains debated. While there is a clear increase in pCR rates with the addition of carboplatin to standard anthracycline-taxane chemotherapy, the long-term benefit of the addition of platinum is less clear^5,6,8,13^. This had led to uneven uptake of carboplatin in clinical practice. Among patients with germline *BRCA* mutations and metastatic breast cancer, the TNT, OlymiAD, and Brocade3 trials showed improved response rate and progression-free survival with the addition of carboplatin and/or PARP inhibitors^9,10,14^. This led to the hypothesis that patients with a germline *BRCA* mutation may specifically benefit from the addition of platinum chemotherapy and/or a PARP inhibitor to NAC. However, in the neoadjuvant setting, despite small studies suggesting a benefit of platinum chemotherapy in patients with *BRCA* mutations^15,16^, GeparSixto and INFORM showed no clear benefit with the addition of carboplatin or cisplatin, respectively^17,18^, reinforcing the need for greater understanding of the different results of metastatic trials versus neoadjuvant trials.

In this study, we present a large cohort of gBRCA patients compared with well-matched non-gBRCA controls, all of whom received uniform therapy as part of a randomized clinical trial consisting of standard NAC alone or with the addition of carboplatin with or without veliparib. This provides an opportunity to investigate the key questions of 1) whether matched patients with germline *BRCA* mutations benefit from the addition of carboplatin and/or PARP inhibitor; and 2) whether tumors with germline *BRCA* mutations have distinct proliferation, immune, and genomic instability gene expression signatures. This study builds on our initial analyses of RNAseq in this cohort that demonstrated carboplatin benefit was not significantly different in basal-like vs. non-basal subgroups but tumors with higher inferred CD8^+^ T-cell infiltration may receive greater benefit with addition of carboplatin in exploratory gene expression immune analyses^11^.

We found no significant difference in pCR between gBRCA and non-gBRCA patients among treatment arms in BrighTNess. This analysis does not include long-term follow up data and, notably, also does not include somatic *BRCA* mutations. We await event-free survival data from the BrighTNess study as well as phase III neoadjuvant trials in early-stage TNBC of NRG-BR003 (NCT024889967) comparing AC-T vs AC-TC as well as NSABP B-56 comparing VCT-AC to TC-AC and T-AC (NCT02032277). However, this cohort establishes that germline *BRCA* mutation carriers with TNBC do not receive further pCR benefit from the addition of carboplatin with or without veliparib to NAC. Intriguingly, the presence of a g*BRCA* mutation did not predict an increase in the pCR rate with the addition of either carboplatin or carboplatin and veliparib to neoadjuvant T-AC chemotherapy; in fact, it was in non-gBRCA patients that the addition of carboplatin with or without veliparib significantly increased the pCR rate. We hypothesize that with homologous recombination deficiency, the gBRCA tumors may be already intrinsically highly sensitive to standard anthracycline-taxane chemotherapy, offering less potential benefit with additional therapy. It should be noted that the data in this study do not have implications for the use of adjuvant PARP inhibitors for gBRCA carriers, related to the recently-published OlympiA study^19^.

The fact that the gBRCA and non-gBRCA cohorts were so well balanced allows for straightforward comparison of pCR in the two groups. However, it is surprising that the gBRCA and non-gBRCA cohorts in this study are so well matched by PAM50 subtype, TNBC subtype, PAM50 proliferation score, GeparSixto score, and proportions of subsets of tumor-infiltrating immune cells. Previous reports have shown that both *BRCA1* and *2* tumors tend to be BL1 and BL2 TNBCtypes, *BRCA1* tumors tend to be basal-like PAM50 subtype with high tumor inflammation signatures and many tumor-infiltrating immune cells, and *BRCA2* tumors tend to be luminal A/B PAM50 subtype^20–25^.

In the cohort of both gBRCA and non-gBRCA patients, higher PAM50 proliferation score, CIN70 score, and GeparSixto immune signature were associated with higher odds of pCR, which is consistent with previous reports of association of pCR with high proliferation and immune signatures in breast cancer^26–29^, but inconsistent with the translational analysis of I-SPY2 which found that CIN70 did not predict response to veliparib with carboplatin^30^. The strong association found between CIN70 and proliferation reflects that the CIN70 score contains genes associated with proliferation and is consistent with CIN70 correlation with tumor grade^31^.

Recently, two studies demonstrate that the addition of immunotherapy to T-AC NAC alone^32^ or plus carboplatin^33^ further enhance pCR rates. As of yet, no subset analyses of germline *BRCA* patients from these studies have been reported. As we seek to personalize therapy for breast cancer patients, further research is warranted into the complex interplay between germline *BRCA* mutation, platinum chemotherapy or PARP inhibitors, and the immune microenvironment and immunotherapy.

This study does have limitations. This is an exploratory secondary analysis of clinical and genomic data from a phase III clinical trial and the power of the analysis may be limited by the small sample size. As these were not prespecified analyses, we pursued a matched cohort study design, which overcomes limitations of gBRCA vs. non-gBRCA studies where receptor subtype are frequently mixed and treatment heterogeneous, and attempted to control for covariates in multivariable analyses. Additionally, DNA metrics of HRD have not yet been fully analyzed (e.g. DNA based LOH scars, RAD51 focus formation, HRDetect mutational signatures), although preliminary analyses of Myriad HRD assay was not associated with differential benefit to addition of carboplatin alone or with veliparib^34^. Veliparib is established to have relatively less PARP trapping relative to other PARP inhibitors, which my facilitate combination with chemotherapy yet also limits our ability to extrapolate to other PARP inhibitors. Finally, recent FDA approval of immunotherapy as part of neoadjuvant therapy for TNBCs in the US reinforces the importance of validation studies in immunotherapy-containing cohorts.

In conclusion, in the overall cohort of both gBRCA and non-gBRCA patients, higher proliferation score, CIN70 score, and GeparSixto immune signature were associated with higher pCR rate and may be more useful biomarkers of patients who will benefit from the addition of carboplatin to neoadjuvant AC-T chemotherapy. The addition of carboplatin or carboplatin and veliparib to neoadjuvant T-AC chemotherapy was associated with increased pCR rate in non-gBRCA patients but not g*BRCA* carriers.

## Methods

### Study population and cohort selection

The BrighTNess trial (NCT02032277, registered January 10, 2014) enrolled 634 patients with stage II/III TNBC and was performed in accordance with The Code of Ethics of the World Medical Association (Declaration of Helsinki). Informed consent was obtained for all human subjects. Patients were randomized 2:1:1 to Arm A: TCV (paclitaxel 80 mg/m ^2^ IV weekly for 12 doses plus carboplatin AUC 6 IV every 3 weeks for four cycles plus veliparib 50 mg orally twice daily for 12 weeks); Arm B: TC (paclitaxel plus carboplatin plus veliparib placebo); or Arm C: T (paclitaxel plus carboplatin placebo plus veliparib placebo). Then all patients received AC every 2–3 weeks for four cycles, with the schedule selected by the treating physician. We matched non-gBRCA patients 2:1 (*N* = 150:75) to gBRCA cases by treatment arm, lymph node stage, and 10-year age range to balance the groups and reduce confounding. This cohort represents 225 of the previously assessed 482 patients^11^. The present analysis was not a preplanned analysis. Clinical data were locked as of January 1, 2018.

### Ethical standards

The trial was performed in accordance with The Code of Ethics of the World Medical Association (Declaration of Helsinki) and was conducted according to the protocol approved by institutional review boards at investigational sites. The full protocol is available as a Supplemental file. Informed consent was obtained for all human subjects. This matched cohort study was approved by the Dana-Farber Cancer Institute Institutional Review Board.

### Whole transcriptome gene expression analyses

A pre-treatment biopsy was collected in RNAlater. As detailed previously^11^, total RNA was extracted and underwent whole transcriptome RNA sequencing (RNAseq) on an Illumina HiSeq 3000 with single end 50 bp reads using RiboZero Gold rRNA depletion at the Washington University McDonnell Genome Institute. Samples with >10 million unique reads were included for further analyses as Reads per Kilobase per Million Reads (RPKM). RNA-seq reads were aligned to the Ensembl release 76 top-level assembly with STAR version 2.0.4b. Gene counts were derived from the number of uniquely aligned unambiguous reads by Subread:featureCount version 1.4.5. Transcript counts were produced by Sailfish version 0.6.3. All gene-level and transcript counts were then imported into the R/Bioconductor package EdgeR and TMM normalization size factors were calculated to adjust samples for differences in library size, resulting in RPKM which were used in downstream analyses. Genes or transcripts not expressed in any sample or less than one count-per-million in the minimum group size minus one were excluded from further analysis.

PAM50 subtype was determined with the ‘Bioclassifier’ package^35^ after balancing TNBC data with an equal number of estrogen receptor-positive cases from The Cancer Genome Atlas^36^. TNBCtype was determined with the TNBCtype tool after normalization to fixed upper quantile^24,37^. PAM50 ‘proliferation signature’ was derived from the ‘Bioclassifier’ package^35^. The GeparSixto immune signature of genes associated with tumor-infiltrating lymphocytes in GeparSixto^38^ and CIN70 signature of chromosomal instability^31^ were calculated as described previously. Proportions of infiltrating immune cell subsets were calculated using the TIMER algorithm^39^. Single sample Gene Set Enrichment Analysis (ssGSEA)^40^ was performed using Hallmark and Immune Response In Silico (IRIS)^12^/ImmuneSigDB^41^ gene sets.

### Statistical analyses

pCR was determined by site pathologists following completion of neoadjuvant therapy, and was defined as absence of cancer cells in the breast and lymph nodes^8^. Chi-square tests were used to compare cohort characteristics. Student’s t-tests were used to compare the mean of continuous variables where the assumption of equal variances was met. For variables that did not meet this assumption, Welch’s t-tests were used. Logistic regression was performed to compare pCR with PAM50 subtype, proliferation score, GeparSixto score, CIN70 score, treatment arm, *BRCA* status, and interactions. T-tests were used to compare ssGSEA gene set scores, with multiple test correction using the method of Benjamini-Hochberg. Statistical analyses were performed with SAS version 9.4. Figures were generated with R version 3.5.1.

### Reporting summary

Further information on research design is available in the Nature Research Reporting Summary linked to this article.

## Supplementary information

Supplementary Information

Reporting Summary

## Data Availability

All raw and processed sequencing files are uploaded and available through restricted access in Alliance Standardized Translational Omics Resource (A-STOR) with accession ASTOR_r6252020. Because the study did not meet submission requirements for dbGaP as a non-NIH funded study, the transcript abundance data, deidentified clinical data, and data dictionary are publicly available on NCBI GEO (accession GSE164458).
