# Supplementary material for: Matched cohort study of germline BRCA mutation carriers with triple negative breast cancer in brightness
Source: NPJ Breast Cancer. 2021 Nov 11;7:142. doi: 10.1038/s41523-021-00349-y (PMC8586340; doi:10.1038/s41523-021-00349-y)
Supplement: Supplementary file 1 — Supplementary Information [file 41523_2021_349_MOESM1_ESM.pdf]

Supplementary Data

Matched Cohort Study of Germline BRCA Mutation Carriers with Triple-Negative Breast Cancer in BrighTNess Trial

Supplementary Figures: 1

Supplementary Tables: 4

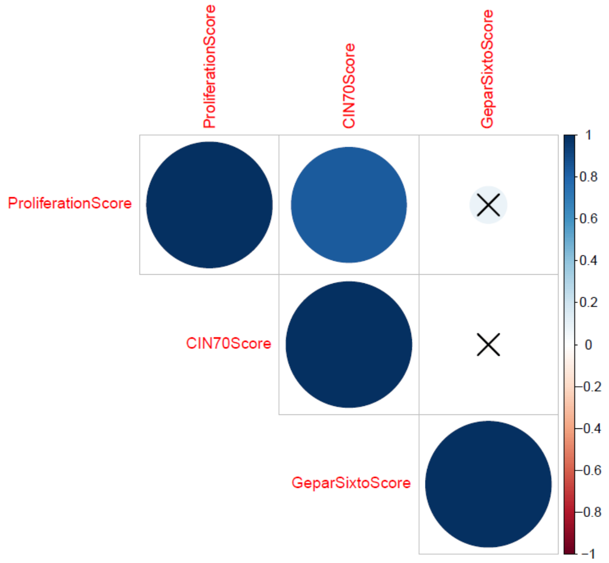

Supplementary Figure 1. Correlation plot for proliferation, CIN70, and GeparSixto scores. Size of circle and darkness of color indicate magnitude of Pearson's r. Blue indicates a positive correlation and red indicates a negative correlation. Non-significant correlations are indicated with an "x".

Supplementary Table 1. *BRCA* status stratified analyses of pathologic complete response by treatment.

	Germline BRCA mutated (gBRCA)		Non-gBRCA	
	N	%	N	%
Arm A	24/39	61.5	47/78	60.3
Arm B	9/18	50.0	23/36	63.9
Arm C	8/18	44.4	9/36	25.0

Supplementary Table 2. ***BRCA*** **status stratified analyses of pathologic complete response by treatment.**

	Germline BRCA mutated (gBRCA)			Non-gBRCA		
	Odds Ratio	95% Confidence Interval	p-value	Odds Ratio	95% Confidence Interval	p-value
Arm A vs. C	2.0	(0.65,6.2)	0.23	4.5	(1.9,11.0)	0.0007
Arm B vs. C	1.3	(0.34,4.6)	0.74	5.3	(1.9,14.7)	0.001

Supplementary Table 3. Single Sample Hallmark Gene Set Enrichment Analysis of Germline BRCA Mutated Cases versus Matched Controls

Signature	T.test_Pvalue	FDR_Pvalue
HALLMARK_WNT_BETA_CATENIN_SIGNALING	0.032812987	0.885565934
HALLMARK_PROTEIN_SECRETION	0.129824967	0.885565934
HALLMARK_ALLOGRAFT_REJECTION	0.137540348	0.885565934
HALLMARK_ESTROGEN_RESPONSE_EARLY	0.150629922	0.885565934
HALLMARK_GLYCOLYSIS	0.179441125	0.885565934
HALLMARK_KRAS_SIGNALING_UP	0.183980092	0.885565934
HALLMARK_APICAL_JUNCTION	0.19917611	0.885565934
HALLMARK_NOTCH_SIGNALING	0.20454539	0.885565934
HALLMARK_COMPLEMENT	0.261703241	0.885565934
HALLMARK_DNA_REPAIR	0.265367764	0.885565934
HALLMARK_TNFA_SIGNALING_VIA_NFKB	0.268393765	0.885565934
HALLMARK_UNFOLDED_PROTEIN_RESPONSE	0.312149705	0.885565934
HALLMARK_INFLAMMATORY_RESPONSE	0.314928077	0.885565934
HALLMARK_SPERMATOGENESIS	0.324627538	0.885565934
HALLMARK_TGF_BETA_SIGNALING	0.33856888	0.885565934
HALLMARK_APOPTOSIS	0.340159966	0.885565934
HALLMARK_ANGIOGENESIS	0.379319253	0.885565934
HALLMARK_CHOLESTEROL_HOMEOSTASIS	0.389575707	0.885565934
HALLMARK_PANCREAS_BETA_CELLS	0.415323297	0.885565934
HALLMARK_COAGULATION	0.430364402	0.885565934
HALLMARK_UV_RESPONSE_DN	0.438149373	0.885565934
HALLMARK_EPITHELIAL_MESENCHYMAL_TRANSITION	0.453465358	0.885565934
HALLMARK_HEME_METABOLISM	0.474087948	0.885565934
HALLMARK_XENOBIOTIC_METABOLISM	0.498728702	0.885565934
HALLMARK_BILE_ACID_METABOLISM	0.50695935	0.885565934
HALLMARK_ESTROGEN_RESPONSE_LATE	0.521187964	0.885565934
HALLMARK_MYOGENESIS	0.543984314	0.885565934
HALLMARK_KRAS_SIGNALING	0.545898788	0.885565934
HALLMARK_MTORC1_SIGNALING	0.551413552	0.885565934
HALLMARK_ANDROGEN_RESPONSE	0.565042417	0.885565934
HALLMARK_PEROXISOME	0.583716397	0.885565934
HALLMARK_MYC_TARGETS_V2	0.604203365	0.885565934
HALLMARK_E2F_TARGETS	0.616297805	0.885565934
HALLMARK_UV_RESPONSE	0.618384249	0.885565934
HALLMARK_HEDGEHOG_SIGNALING	0.640043188	0.885565934
HALLMARK_PI3K_AKT_MTOR_SIGNALING	0.64682362	0.885565934
HALLMARK_OXIDATIVE_PHOSPHORYLATION	0.653270694	0.885565934
HALLMARK_INTERFERON_GAMMA_RESPONSE	0.675133373	0.885565934
HALLMARK_APICAL_SURFACE	0.679193238	0.885565934
HALLMARK_KRAS_SIGNALING_DN	0.681204564	0.885565934
HALLMARK_IL2_STAT5_SIGNALING	0.703781314	0.887906098
HALLMARK_FATTY_ACID_METABOLISM	0.717154925	0.887906098
HALLMARK_P53_PATHWAY	0.746258372	0.902451985
HALLMARK_HYPOXIA	0.770993075	0.911173634
HALLMARK_G2M_CHECKPOINT	0.824588226	0.952857506
HALLMARK_MYC_TARGETS_V1	0.853475994	0.96479895
HALLMARK_INTERFERON_ALPHA_RESPONSE	0.904727096	0.968350969
HALLMARK_MITOTIC_SPINDLE	0.913879961	0.968350969
HALLMARK_UV_RESPONSE_UP	0.92300621	0.968350969
HALLMARK_REACTIVE_OXIGEN_SPECIES_PATHWAY	0.943799376	0.968350969
HALLMARK_IL6_JAK_STAT3_SIGNALING	0.952125237	0.968350969
HALLMARK_ADIPOGENESIS	0.968350969	0.968350969

Supplementary Table 4. Single Sample Immune Response In Silico Gene Set Enrichment Analysis of Germline BRCA Mutated Cases versus Matched Controls

Signature	T.test_Pvalue	FDR_Pvalue
GSE22886_NEUTROPHIL_VS_DC	0.052923328	0.992449816
GSE22886_NAIVE_VS_IGM_MEMORY_BCELL	0.054589297	0.992449816
GSE22886_NEUTROPHIL_VS_DC_UP	0.067489042	0.992449816
GSE22886_IGG_IGA_MEMORY_BCELL_VS_BM_PLASMA_CELL_UP	0.082740987	0.992449816
GSE22886_NAIVE_VS_IGM_MEMORY_BCELL_UP	0.084395373	0.992449816
GSE22886_IGM_MEMORY_BCELL_VS_BM_PLASMA_CELL_UP	0.10910867	0.992449816
GSE22886_DAY0_VS_DAY7_MONOCYTE_IN_CULTURE_UP	0.11180083	0.992449816
GSE22886_NAIVE_CD8_TCELL_VS_NKCELL	0.123627711	0.992449816
GSE22886_NAIVE_CD4_TCELL_VS_MONOCYTE_UP	0.155678174	0.992449816
GSE22886_NAIVE_BCELL_VS_MONOCYTE_DN	0.158466133	0.992449816
GSE22886_NAIVE_CD4_TCELL_VS_MONOCYTE_DN	0.159572469	0.992449816
GSE22886_NAIVE_BCELL_VS_MONOCYTE_UP	0.159743287	0.992449816
GSE22886_IGA_VS_IGM_MEMORY_BCELL	0.163567329	0.992449816
GSE22886_DC_VS_MONOCYTE_DN	0.163684792	0.992449816
GSE22886_IGA_VS_IGM_MEMORY_BCELL_DN	0.169889527	0.992449816
GSE22886_DAY0_VS_DAY1_MONOCYTE_IN_CULTURE_DN	0.196352045	0.992449816
GSE22886_NAIVE_TCELL_VS_MONOCYTE_DN	0.196617726	0.992449816
GSE22886_NAIVE_CD8_TCELL_VS_MONOCYTE_DN	0.202673149	0.992449816
GSE22886_NAIVE_VS_IGM_MEMORY_BCELL_DN	0.208200895	0.992449816
GSE22886_DC_VS_MONOCYTE	0.210527101	0.992449816
GSE22886_NAIVE_CD8_TCELL_VS_NKCELL_DN	0.213687281	0.992449816
GSE22886_IGG_IGA_MEMORY_BCELL_VS_BLOOD_PLASMA_CELL_DN	0.215485743	0.992449816
GSE22886_NAIVE_CD4_TCELL_VS_48H_ACT_TH2_UP	0.236004937	0.992449816
GSE22886_NAIVE_CD8_TCELL_VS_NKCELL_UP	0.242888831	0.992449816
GSE22886_IGM_MEMORY_BCELL_VS_BLOOD_PLASMA_CELL_UP	0.258253176	0.992449816
GSE22886_TH1_VS_TH2_48H_ACT	0.261509237	0.992449816
GSE22886_NAIVE_BCELL_VS_BLOOD_PLASMA_CELL_DN	0.266970882	0.992449816
GSE22886_IGM_MEMORY_BCELL_VS_BLOOD_PLASMA_CELL_DN	0.269544809	0.992449816
GSE22886_NAIVE_CD8_TCELL_VS_MEMORY_TCELL_DN	0.270795456	0.992449816
GSE22886_DAY1_VS_DAY7_MONOCYTE_IN_CULTURE_UP	0.27225891	0.992449816
GSE22886_IGG_IGA_MEMORY_BCELL_VS_BM_PLASMA_CELL	0.276167255	0.992449816
GSE22886_NAIVE_CD8_TCELL_VS_DC_DN	0.278824442	0.992449816
GSE22886_NAIVE_TCELL_VS_NKCELL	0.290298865	0.992449816
GSE22886_NAIVE_BCELL_VS_NEUTROPHIL_DN	0.300482633	0.992449816
GSE22886_NAIVE_TCELL_VS_NKCELL_DN	0.301222491	0.992449816
GSE22886_CD8_VS_CD4_NAIVE_TCELL	0.305766133	0.992449816
GSE22886_IGM_MEMORY_BCELL_VS_BM_PLASMA_CELL	0.307041001	0.992449816
GSE22886_CD8_VS_CD4_NAIVE_TCELL_DN	0.307268086	0.992449816
GSE22886_NAIVE_BCELL_VS_DC_DN	0.309211881	0.992449816
GSE22886_TH1_VS_TH2_48H_ACT_DN	0.315130595	0.992449816
GSE22886_DAY0_VS_DAY7_MONOCYTE_IN_CULTURE_DN	0.321778593	0.992449816
GSE22886_NAIVE_BCELL_VS_NEUTROPHIL	0.330739403	0.992449816
GSE22886_IGG_IGA_MEMORY_BCELL_VS_BLOOD_PLASMA_CELL_UP	0.331489827	0.992449816
GSE22886_NAIVE_TCELL_VS_DC_DN	0.348013355	0.992449816
GSE22886_TH1_VS_TH2_48H_ACT_UP	0.351564905	0.992449816
GSE22886_NEUTROPHIL_VS_MONOCYTE_DN	0.365753413	0.992449816
GSE22886_IL2_VS_IL15_STIM_NKCELL_DN	0.37124705	0.992449816
GSE22886_NAIVE_VS_IGG_IGA_MEMORY_BCELL_DN	0.371404648	0.992449816
GSE22886_NAIVE_VS_MEMORY_TCELL_DN	0.371950576	0.992449816
GSE22886_DAY0_VS_DAY1_MONOCYTE_IN_CULTURE_UP	0.374706168	0.992449816
GSE22886_UNSTIM_VS_IL2_STIM_NKCELL_UP	0.376114361	0.992449816
GSE22886_UNSTIM_VS_STIM_MEMORY_TCELL_UP	0.385321281	0.992449816
GSE22886_DAY0_VS_DAY7_MONOCYTE_IN_CULTURE	0.392327887	0.992449816
GSE22886_NAIVE_CD8_TCELL_VS_MONOCYTE_UP	0.393498184	0.992449816
GSE22886_IL2_VS_IL15_STIM_NKCELL	0.45367746	0.992449816
GSE22886_DAY1_VS_DAY7_MONOCYTE_IN_CULTURE	0.481577593	0.992449816
GSE22886_CD8_VS_CD4_NAIVE_TCELL_UP	0.482146879	0.992449816
GSE22886_NAIVE_TCELL_VS_NKCELL_UP	0.485796745	0.992449816
GSE22886_TH1_VS_TH2_12H_ACT_DN	0.494942278	0.992449816
GSE22886_IGA_VS_IGM_MEMORY_BCELL_UP	0.498654962	0.992449816
GSE22886_TH1_VS_TH2_12H_ACT_UP	0.525123151	0.992449816
GSE22886_NEUTROPHIL_VS_MONOCYTE	0.533564006	0.992449816
GSE22886_UNSTIM_VS_IL2_STIM_NKCELL	0.545351067	0.992449816
GSE22886_NAIVE_CD4_TCELL_VS_MEMORY_TCELL_DN	0.556298937	0.992449816
GSE22886_NAIVE_CD4_TCELL_VS_12H_ACT_TH1_UP	0.569691311	0.992449816
GSE22886_NAIVE_TCELL_VS_MONOCYTE_UP	0.570420024	0.992449816
GSE22886_NAIVE_CD4_TCELL_VS_DC_DN	0.571127765	0.992449816
GSE22886_NAIVE_TCELL_VS_DC	0.584264304	0.992449816
GSE22886_TCELL_VS_BCELL_NAIVE_UP	0.58536891	0.992449816
GSE22886_NAIVE_BCELL_VS_BLOOD_PLASMA_CELL_UP	0.593875382	0.992449816
GSE22886_NAIVE_CD8_TCELL_VS_DC	0.598457546	0.992449816
GSE22886_NAIVE_TCELL_VS_MONOCYTE	0.607512536	0.992449816
GSE22886_NAIVE_CD4_TCELL_VS_NKCELL_DN	0.610157911	0.992449816
GSE22886_NAIVE_CD4_TCELL_VS_NKCELL_UP	0.617108649	0.992449816
GSE22886_DAY0_VS_DAY1_MONOCYTE_IN_CULTURE	0.620046659	0.992449816

Supplementary Table 4. (cont)

GSE22886_DAY1_VS_DAY7_MONOCYTE_IN_CULTURE_DN	0.627523773	0.992449816
GSE22886_NAIVE_VS_MEMORY_TCELL	0.631563883	0.992449816
GSE22886_NAIVE_CD8_TCELL_VS_MEMORY_TCELL_UP	0.63579439	0.992449816
GSE22886_NAIVE_CD4_TCELL_VS_12H_ACT_TH2_UP	0.643180147	0.992449816
GSE22886_NAIVE_VS_IGG_IGA_MEMORY_BCELL	0.646914836	0.992449816
GSE22886_IL2_VS_IL15_STIM_NKCELL_UP	0.669628092	0.992449816
GSE22886_NAIVE_CD8_TCELL_VS_MONOCYTE	0.673784239	0.992449816
GSE22886_CD8_TCELL_VS_BCELL_NAIVE_UP	0.680313357	0.992449816
GSE22886_TH1_VS_TH2_12H_ACT	0.681063091	0.992449816
GSE22886_NEUTROPHIL_VS_DC_DN	0.683949548	0.992449816
GSE22886_NAIVE_CD4_TCELL_VS_MEMORY_TCELL	0.693579173	0.992449816
GSE22886_CD8_TCELL_VS_BCELL_NAIVE_DN	0.699570967	0.992449816
GSE22886_NEUTROPHIL_VS_MONOCYTE_UP	0.714083125	0.992449816
GSE22886_NAIVE_BCELL_VS_BM_PLASMA_CELL_UP	0.715192421	0.992449816
GSE22886_NAIVE_CD4_TCELL_VS_12H_ACT_TH1_DN	0.721106028	0.992449816
GSE22886_NAIVE_CD4_TCELL_VS_48H_ACT_TH2_DN	0.727423558	0.992449816
GSE22886_NAIVE_BCELL_VS_BLOOD_PLASMA_CELL	0.731413105	0.992449816
GSE22886_NAIVE_BCELL_VS_MONOCYTE	0.731770105	0.992449816
GSE22886_NAIVE_CD4_TCELL_VS_NEUTROPHIL_DN	0.740628765	0.992449816
GSE22886_UNSTIM_VS_IL15_STIM_NKCELL_UP	0.757275457	0.992449816
GSE22886_NAIVE_CD4_TCELL_VS_12H_ACT_TH2_DN	0.765163601	0.992449816
GSE22886_CD4_TCELL_VS_BCELL_NAIVE_DN	0.771227266	0.992449816
GSE22886_UNSTIM_VS_STIM_MEMORY_TCELL_DN	0.774770783	0.992449816
GSE22886_CD4_TCELL_VS_BCELL_NAIVE_UP	0.777955657	0.992449816
GSE22886_NAIVE_CD8_TCELL_VS_DC_UP	0.785145413	0.992449816
GSE22886_NAIVE_CD4_TCELL_VS_DC_UP	0.79313471	0.992449816
GSE22886_UNSTIM_VS_IL15_STIM_NKCELL	0.79621323	0.992449816
GSE22886_NAIVE_CD4_TCELL_VS_48H_ACT_TH2	0.800036298	0.992449816
GSE22886_IGG_IGA_MEMORY_BCELL_VS_BM_PLASMA_CELL_DN	0.801434496	0.992449816
GSE22886_NAIVE_CD8_TCELL_VS_MEMORY_TCELL	0.813146865	0.992449816
GSE22886_NAIVE_BCELL_VS_DC	0.815379961	0.992449816
GSE22886_UNSTIM_VS_IL2_STIM_NKCELL_DN	0.815887739	0.992449816
GSE22886_NAIVE_CD4_TCELL_VS_NEUTROPHIL	0.819524958	0.992449816
GSE22886_DC_VS_MONOCYTE_UP	0.824702796	0.992449816
GSE22886_CTRL_VS_LPS_24H_DC_DN	0.826549419	0.992449816
GSE22886_NAIVE_CD4_TCELL_VS_NEUTROPHIL_UP	0.828791945	0.992449816
GSE22886_TCELL_VS_BCELL_NAIVE_DN	0.828868233	0.992449816
GSE22886_TCELL_VS_BCELL_NAIVE	0.830486634	0.992449816
GSE22886_NAIVE_CD8_TCELL_VS_NEUTROPHIL_UP	0.83478815	0.992449816
GSE22886_NAIVE_CD8_TCELL_VS_NEUTROPHIL	0.841330774	0.992449816
GSE22886_NAIVE_VS_IGG_IGA_MEMORY_BCELL_UP	0.841825266	0.992449816
GSE22886_UNSTIM_VS_IL15_STIM_NKCELL_DN	0.845942673	0.992449816
GSE22886_NAIVE_BCELL_VS_DC_UP	0.854049994	0.992449816
GSE22886_CTRL_VS_LPS_24H_DC	0.856014845	0.992449816
GSE22886_NAIVE_BCELL_VS_BM_PLASMA_CELL	0.857654173	0.992449816
GSE22886_NAIVE_CD4_TCELL_VS_MEMORY_TCELL_UP	0.867467039	0.992449816
GSE22886_NAIVE_TCELL_VS_NEUTROPHIL	0.872201142	0.992449816
GSE22886_NAIVE_CD4_TCELL_VS_DC	0.887160376	0.992449816
GSE22886_CTRL_VS_LPS_24H_DC_UP	0.887367677	0.992449816
GSE22886_IGM_MEMORY_BCELL_VS_BM_PLASMA_CELL_DN	0.899821898	0.992449816
GSE22886_NAIVE_TCELL_VS_DC_UP	0.901902106	0.992449816
GSE22886_IGM_MEMORY_BCELL_VS_BLOOD_PLASMA_CELL	0.902617	0.992449816
GSE22886_NAIVE_TCELL_VS_NEUTROPHIL_DN	0.902995724	0.992449816
GSE22886_IGG_IGA_MEMORY_BCELL_VS_BLOOD_PLASMA_CELL	0.906117199	0.992449816
GSE22886_NAIVE_TCELL_VS_NEUTROPHIL_UP	0.910470695	0.992449816
GSE22886_NAIVE_CD4_TCELL_VS_NKCELL	0.912287352	0.992449816
GSE22886_NAIVE_CD8_TCELL_VS_NEUTROPHIL_DN	0.912333886	0.992449816
GSE22886_UNSTIM_VS_STIM_MEMORY_TCELL	0.917427863	0.992449816
GSE22886_NAIVE_BCELL_VS_NEUTROPHIL_UP	0.931295021	0.992449816
GSE22886_NAIVE_CD4_TCELL_VS_48H_ACT_TH1_UP	0.944653849	0.992449816
GSE22886_NAIVE_CD4_TCELL_VS_12H_ACT_TH1	0.96506936	0.992449816
GSE22886_NAIVE_CD4_TCELL_VS_MONOCYTE	0.970876549	0.992449816
GSE22886_NAIVE_CD4_TCELL_VS_48H_ACT_TH1	0.976378034	0.992449816
GSE22886_NAIVE_CD4_TCELL_VS_12H_ACT_TH2	0.976935003	0.992449816
GSE22886_NAIVE_VS_MEMORY_TCELL_UP	0.981892419	0.992449816
GSE22886_NAIVE_BCELL_VS_BM_PLASMA_CELL_DN	0.982458439	0.992449816
GSE22886_CD8_TCELL_VS_BCELL_NAIVE	0.989432627	0.992449816
GSE22886_CD4_TCELL_VS_BCELL_NAIVE	0.991033676	0.992449816
GSE22886_NAIVE_CD4_TCELL_VS_48H_ACT_TH1_DN	0.992449816	0.992449816
