# Supplementary material for: Matched cohort study of germline BRCA mutation carriers with triple negative breast cancer in brightness
Source: NPJ Breast Cancer. 2021 Nov 11;7:142. doi: 10.1038/s41523-021-00349-y (PMC8586340; doi:10.1038/s41523-021-00349-y)
Supplement: Supplementary file 2 — Reporting Summary [file 41523_2021_349_MOESM2_ESM.pdf]

nature portfolio

Corresponding author(s): Daniel G. Stover

Last updated by author(s): Sep 1, 2021

Reporting Summary

Nature Portfolio wishes to improve the reproducibility of the work that we publish. This form provides structure for consistency and transparency in reporting. For further information on Nature Portfolio policies, see our Editorial Policies and the Editorial Policy Checklist.

Statistics

For all statistical analyses, confirm that the following items are present in the figure legend, table legend, main text, or Methods section.

n/a	Confirmed	
☐	☒	The exact sample size (*n*) for each experimental group/condition, given as a discrete number and unit of measurement
☐	☒	A statement on whether measurements were taken from distinct samples or whether the same sample was measured repeatedly
☐	☒	The statistical test(s) used AND whether they are one- or two-sided<br>*Only common tests should be described solely by name; describe more complex techniques in the Methods section.*
☐	☒	A description of all covariates tested
☐	☒	A description of any assumptions or corrections, such as tests of normality and adjustment for multiple comparisons
☐	☒	A full description of the statistical parameters including central tendency (e.g. means) or other basic estimates (e.g. regression coefficient) AND variation (e.g. standard deviation) or associated estimates of uncertainty (e.g. confidence intervals)
☐	☒	For null hypothesis testing, the test statistic (e.g. *F*, *t*, *r*) with confidence intervals, effect sizes, degrees of freedom and *P* value noted<br>*Give P values as exact values whenever suitable.*
☒	☐	For Bayesian analysis, information on the choice of priors and Markov chain Monte Carlo settings
☒	☐	For hierarchical and complex designs, identification of the appropriate level for tests and full reporting of outcomes
☒	☐	Estimates of effect sizes (e.g. Cohen's *d*, Pearson's *r*), indicating how they were calculated

Our web collection on statistics for biologists contains articles on many of the points above.

Software and code

Policy information about availability of computer code

Data collection	RNA-seq reads were aligned to the Ensembl release 76 top-level assembly with STAR version 2.0.4b. Gene counts were derived from the number of uniquely aligned unambiguous reads by Subread:featureCount version 1.4.5. Transcript counts were produced by Sailfish version 0.6.3. All gene-level and transcript counts were then imported into the R/Bioconductor package EdgeR and TMM normalization size factors were calculated to adjust samples for differences in library size, resulting in RPKM which were used in downstream analyses.
Data analysis	PAM50 subtype was determined with the 'Bioclassifier' package35 after balancing TNBC data with an equal number of estrogen receptor-positive cases from The Cancer Genome Atlas.36 TNBCtype was determined with the TNBCtype tool after normalization to fixed upper quantile.24,37 PAM50 'proliferation signature' was derived from the 'Bioclassifier' package.35 The GeparSixto immune signature of genes associated with tumor-infiltrating lymphocytes in GeparSixto38 and CIN70 signature of chromosomal instability31 were calculated as described previously. Proportions of infiltrating immune cell subsets were calculated using the TIMER algorithm.39 Single sample Gene Set Enrichment Analysis (ssGSEA)40 was performed using Hallmark and Immune Response In Silico (IRIS)12/ImmuneSigDB41 gene sets.

For manuscripts utilizing custom algorithms or software that are central to the research but not yet described in published literature, software must be made available to editors and reviewers. We strongly encourage code deposition in a community repository (e.g. GitHub). See the Nature Portfolio guidelines for submitting code & software for further information.

Data

Policy information about availability of data

All manuscripts must include a data availability statement. This statement should provide the following information, where applicable:

- Accession codes, unique identifiers, or web links for publicly available datasets
- A description of any restrictions on data availability
- For clinical datasets or third party data, please ensure that the statement adheres to our policy

Data Availability: All raw and processed sequencing files are uploaded and available through restricted access in Alliance Standardized Translational Omics Resource (A-STOR) with accession ASTOR_r6252020. Because the study did not meet submission
requirements for dbGaP as a non-NIH funded study, the transcript abundance data, deidentified clinical data, and data dictionary are publicly available on NCBI GEO (accession GSE164458); https://www.ncbi.nlm.nih.gov/geo/query/acc.cgi?acc=GSE164458.

Field-specific reporting

Please select the one below that is the best fit for your research. If you are not sure, read the appropriate sections before making your selection.

☒ Life sciences ☐ Behavioural & social sciences ☐ Ecological, evolutionary & environmental sciences

For a reference copy of the document with all sections, see nature.com/documents/nr-reporting-summary-flat.pdf

Life sciences study design

All studies must disclose on these points even when the disclosure is negative.

Sample size	The BrighTNess trial (NCT02032277, registered January 10, 2014) enrolled 634 patients with stage II/III TNBC. We matched non-gBRCA patients 2:1 (N= 150:75) to gBRCA cases by treatment arm, lymph node stage, and 10-year age range to balance the groups and reduce confounding.
Data exclusions	Patients who did have tumors available for RNAseq (n=146) or did not complete RNAseq due to quality (n=8) were excluded.
Replication	Analyses were performed once using available data.
Randomization	N/A
Blinding	N/A

Reporting for specific materials, systems and methods

We require information from authors about some types of materials, experimental systems and methods used in many studies. Here, indicate whether each material, system or method listed is relevant to your study. If you are not sure if a list item applies to your research, read the appropriate section before selecting a response.

Materials & experimental systems

n/a	Involved in the study	
☒	☐	Antibodies
☒	☐	Eukaryotic cell lines
☒	☐	Palaeontology and archaeology
☒	☐	Animals and other organisms
☐	☒	Human research participants
☐	☒	Clinical data
☐	☐	Dual use research of concern

Methods

n/a	Involved in the study	
☒	☐	ChIP-seq
☒	☐	Flow cytometry
☒	☐	MRI-based neuroimaging

Human research participants

Policy information about studies involving human research participants

Population characteristics	The BrighTNess trial (NCT02032277, registered January 10, 2014) enrolled 634 patients with stage II/III TNBC and was performed in accordance with The Code of Ethics of the World Medical Association (Declaration of Helsinki). Informed consent was obtained for all human subjects. Patients were randomized 2:1:1 to Arm A: TCV (paclitaxel 80 mg/m 2 IV weekly for 12 doses plus carboplatin AUC 6 IV every 3 weeks for four cycles plus veliparib 50 mg orally twice daily for 12 weeks); Arm B: TC (paclitaxel plus carboplatin plus veliparib placebo); or Arm C: T (paclitaxel plus carboplatin placebo plus veliparib placebo). Then all patients received AC every 2–3 weeks for four cycles, with the schedule selected by the treating physician.

	We matched non-gBRCA patients 2:1 (N= 150:75) to gBRCA cases by treatment arm, lymph node stage, and 10-year age range to balance the groups and reduce confounding. This cohort represents 225 of the previously assessed 482 patients.1
Recruitment	No patients were recruited - secondary analysis of existing data
Ethics oversight	The trial was performed in accordance with The Code of Ethics of the World Medical Association (Declaration of Helsinki) and was conducted according to the protocol approved by institutional review boards at investigational sites. The full protocol is available as a Supplemental file. Informed consent was obtained for all human subjects. This matched cohort study was approved by the Dana-Farber Cancer Institute Institutional Review Board.

Note that full information on the approval of the study protocol must also be provided in the manuscript.

Clinical data

Policy information about clinical studies

All manuscripts should comply with the ICMJE guidelines for publication of clinical research and a completed CONSORT checklist must be included with all submissions.

Clinical trial registration	NCT02032277
Study protocol	Submitted
Data collection	Secondary analyses of existing data
Outcomes	Pathologic complete response versus residual disease

Dual use research of concern

Policy information about dual use research of concern

Hazards

Could the accidental, deliberate or reckless misuse of agents or technologies generated in the work, or the application of information presented in the manuscript, pose a threat to:

No	Yes	
☒	☐	Public health
☒	☐	National security
☒	☐	Crops and/or livestock
☒	☐	Ecosystems
☒	☐	Any other significant area

Experiments of concern

Does the work involve any of these experiments of concern:

No	Yes	
☒	☐	Demonstrate how to render a vaccine ineffective
☒	☐	Confer resistance to therapeutically useful antibiotics or antiviral agents
☒	☐	Enhance the virulence of a pathogen or render a nonpathogen virulent
☒	☐	Increase transmissibility of a pathogen
☒	☐	Alter the host range of a pathogen
☒	☐	Enable evasion of diagnostic/detection modalities
☒	☐	Enable the weaponization of a biological agent or toxin
☒	☐	Any other potentially harmful combination of experiments and agents
